# Supplementary material for: Structure-based discovery of dual pathway inhibitors for SARS-CoV-2 entry
Source: Nat Commun. 2023 Nov 21;14:7574. doi: 10.1038/s41467-023-42527-5 (PMC10663540; doi:10.1038/s41467-023-42527-5)
Supplement: Supplementary file 1 — Supplementary Information [file 41467_2023_42527_MOESM1_ESM.pdf]

Supplementary information for

Structure-based discovery of dual pathway inhibitors for SARS-CoV-2 entry

Haofeng Wang[1,2,12], Qi Yang[3,12], Xiaoce Liu[1,2,12], Zili Xu[4,5,12], Maolin Shao[1,2,12], Dongxu Li[1,2], Yinkai Duan[1,2], Jielin Tang[3], Xianqiang Yu[1,5], Yumin Zhang[6], Aihua Hao[7], Yajie Wang[7], Jie Chen[1,2], Chenghao Zhu[1], Luke Guddat[8], Hongli Chen[1,5], Leike Zhang[6,*], Xinwen Chen[3,*], Biao Jiang[1,5,*], Lei Sun[7,*], Zihe Rao[1,2,3,9,10,11], Haitao Yang[1,2,*]

[*]Correspondence: zhangleike@wh.iov.cn (L.Z.), jiangbiao@shanghaitech.edu.cn (B.J.), chen_xinwen@gzlab.ac.cn (X.C.), llsun@fudan.edu.cn (L.S.), yanght@shanghaitech.edu.cn (H.Y.)

This PDF file includes:

Supplementary Figs. 1-13 and Supplementary Tables 1-3

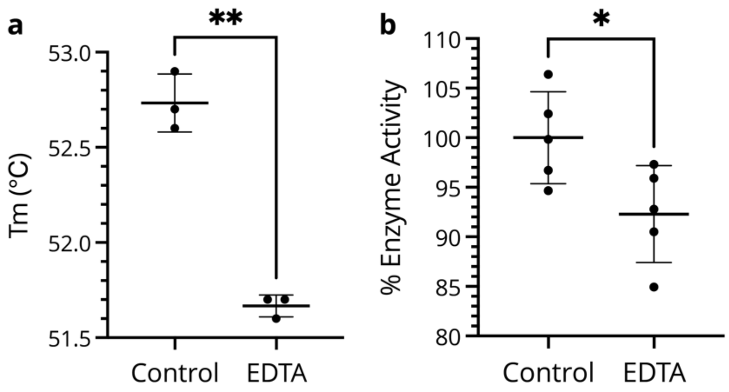

Supplementary Figure. 1. Calcium chelation of LDLRA domain contributes to the stability of TMPRSS2 ectodomain and its enzymatic activity. (a) The thermal stability of TMPRSS2 slightly decreased due to the loss of calcium. **p = 0.0030, two-sided t-test with Welch's correction. (b) The enzymatic activity of TMPRSS2 demonstrated a modest decrease in the absence of calcium. *p = 0.0339, two-sided t-test with Welch's correction.

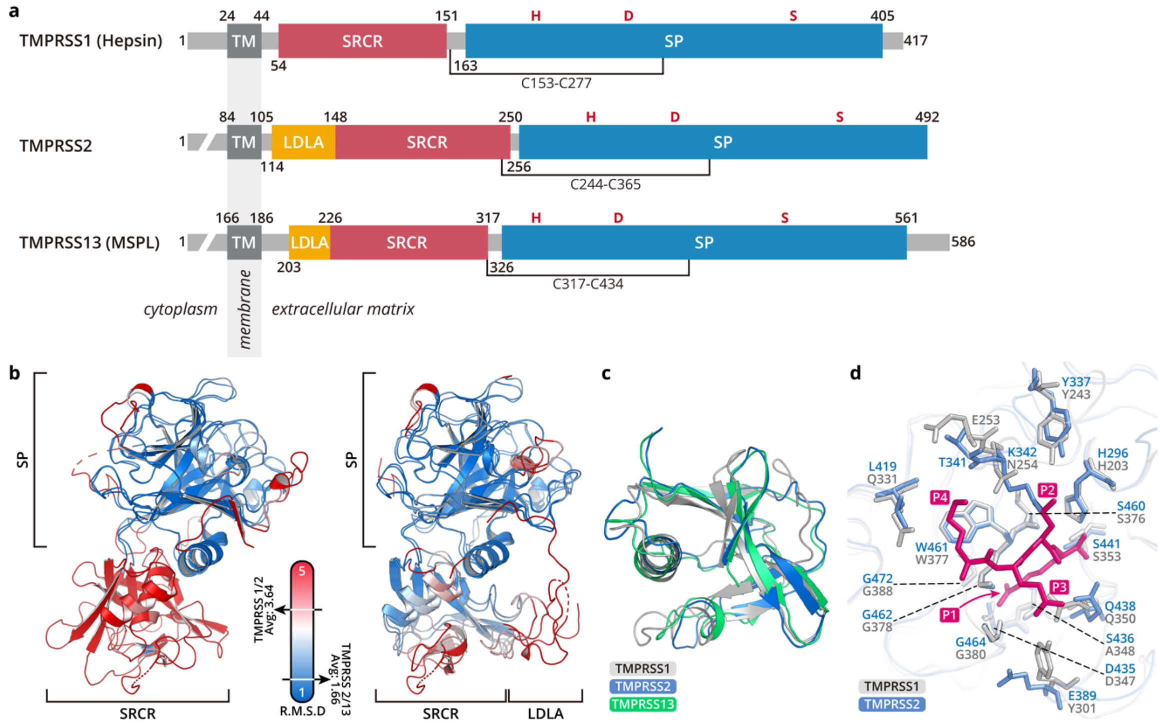

Supplementary Figure 2. Architecture and structural comparison of TMPRSS1, 2, and 13. (a) Schematic architecture of TMPRSS1, 2, and 13. (b) Structural comparison of TMPRSS2 with TMPRSS1 and TMPRSS2 with TMPRSS13. The color spectrum represents the r.m.s.d. of the aligned Cα atoms. (c) The overlay of the SRCR domain of TMPRSS1, 2, and 13. (d) Comparison of the catalytic pocket of TMPRSS2 and TMPRSS1 in complex with tetrapeptide PS-SCL (1Z8G), residues involved in pocket formation were shown as sticks, tetrapeptide PS-SCL is shown as pink sticks.

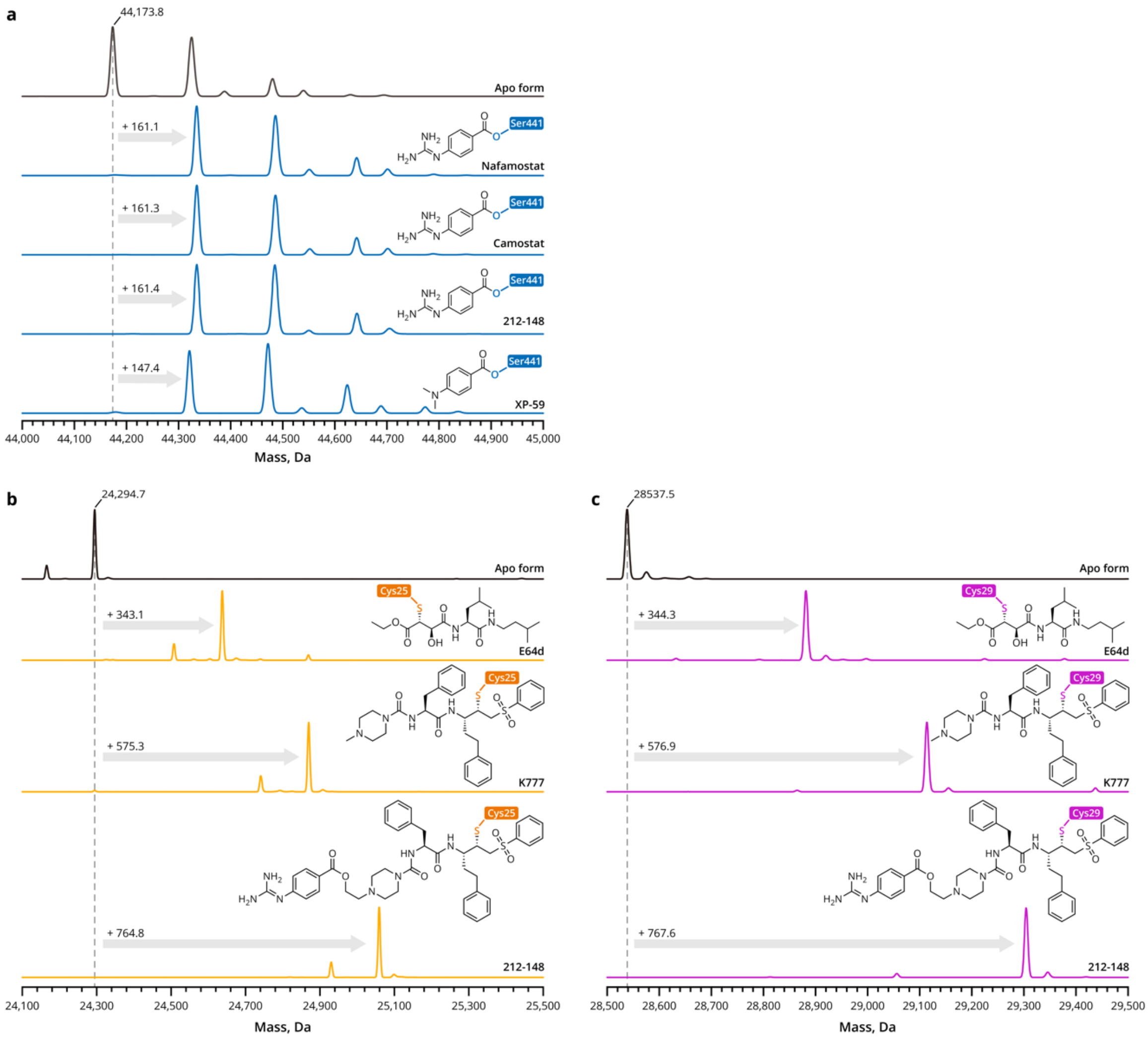

Supplementary Figure 3. Mass spectrum data confirmed that all covalent inhibitors are specifically linked to their target proteases.

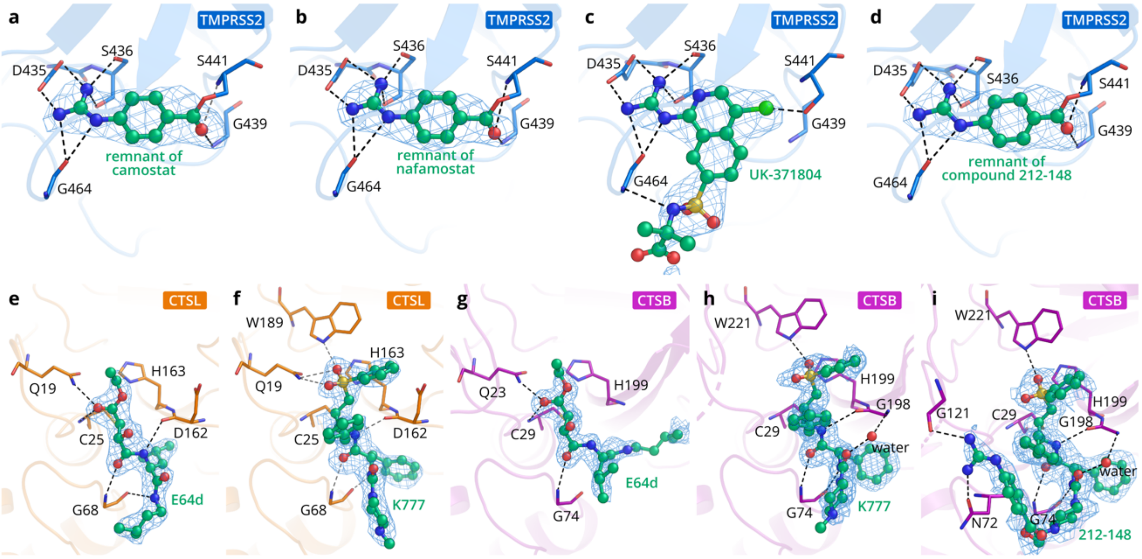

Supplementary Figure 4. Fo-Fc omit map of inhibitors in the complex structures. Crystal structures of TMPRSS2 in complex with camostat (a), nafamostat (b), UK-371804 (c), and 212-148 (d), CTSL in complex with E64d (e) and K777 (f), CTSB in complex with E64d (g), K777 (h), 212-148 (i) are shown, the omit map of the compounds contoured at 2.5σ were shown as blue mesh except for panel i which was contoured at 1.5σ.

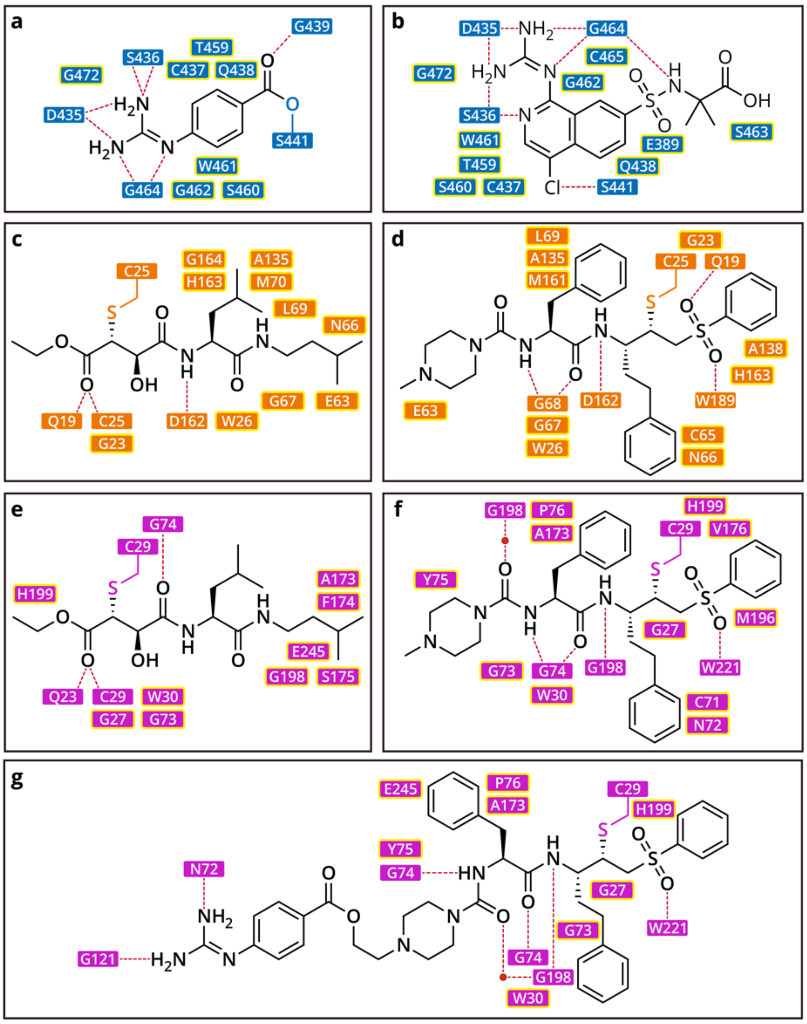

Supplementary Figure 5. Interactions between inhibitors and target proteins. Residues interacting with the compounds are indicated. Hydrogen and ionic bonds are shown as red dashes.

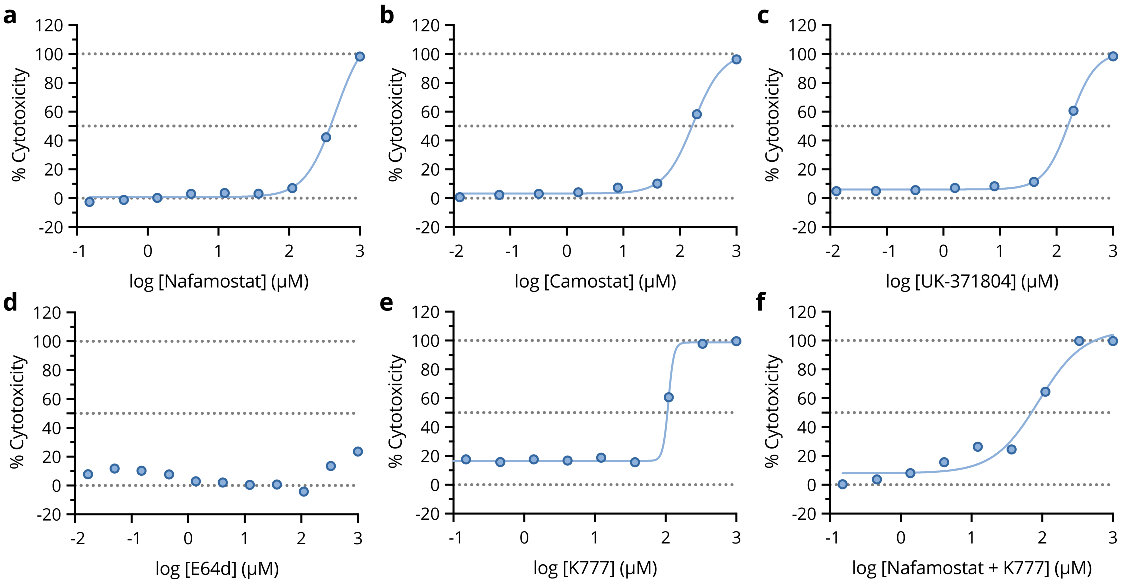

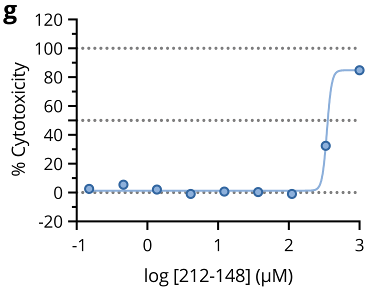

h

		Antiviral efficacy			
Compound	Cell line	SARS-CoV-2 strain	Value (nM)	CC50 (μM) (95% CI)	SI
Nafamostat	Calu-3	Delta	26.3	451.9 (376.0 to 543.2)	17,183
Camostat	Calu-3	Delta	109.1	172.9 (161.0 to 185.7)	1,585
UK-371804	Calu-3	Delta	424.6	171.9 (165.8 to 178.1)	405
E64d	Calu-3	Omicron BA.2	239.8	> 1,000	> 4,000
K777	Calu-3	Delta	638.5	109.6 (36.5 to 328.8)	172
		Omicron BA.2	14.0		7,829
Nafamostat + K777 (1:1)	Calu-3	Delta	0.9	88.6 (65.8 to 119.5)	98,444
212-148	Calu-3	Delta	235.2	347.2 (115.7 to 1041.6)	1,476
		Omicron BA.2	64.4		5,391

Supplementary Figure 6. Cytotoxicity studies. Cytotoxicity of the nafamostat (a), camostat (b), UK-371804 (c), E64d (d), K777 (e), nafamostat + K777 (f), and 212-148 (g) in Calu-3 cells was measured by a Cell Titer-Glo Luminescent Cell Viability assay. The experiments were done in triplicate. (h) A summary of the assay results; SI = CC_{50}/EC_{50}.

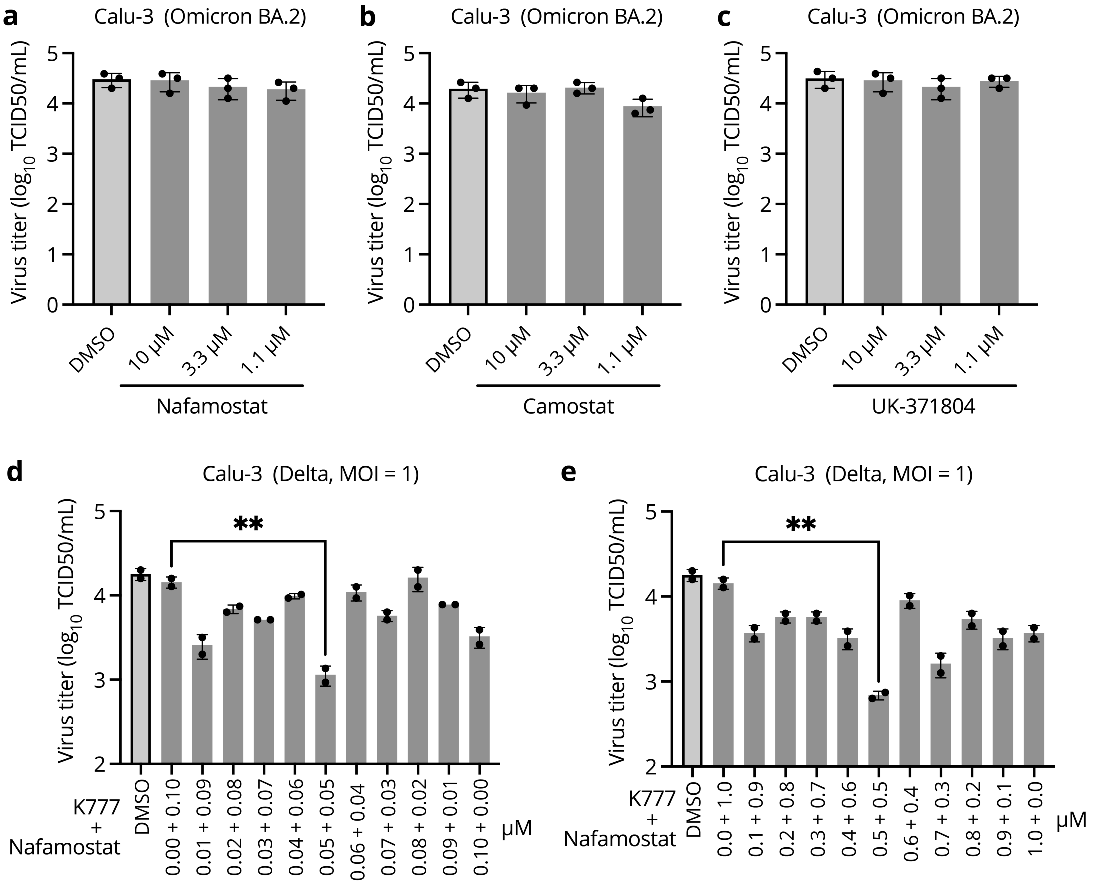

Supplementary Figure 7. Prominent antiviral effect of nafamostat and K777 to inhibit SARS-CoV-2 Delta variants infections. Calu-3 cells were pre-treated with nafamostat (a), camostat (b), and UK-371804 (c) at 1.1 μM, 3.3 μM and 10 μM for 1 hour and then infected with a clinical SARS-CoV-2 isolate omicron strain BA.2 (MOI = 0.1). Twenty-four hours after inoculation, the supernatants were collected and virus titers were determined as $TCID_{50}$/mL. The experiments were performed in triplicate. Data are shown as mean ± S.D. (d-e) Calu-3 cells were pre-treated with K777 and nafamostat at different concentrations for 1 hour and then infected with a clinical SARS-CoV-2 isolate strain Delta (MOI = 1). Twenty-four hours after inoculation, the supernatants were collected and virus titers were determined as $TCID_{50}$/mL. The data are presented as the means ± S.D. **$p < 0.01$, one-way ANOVA. Two independent experiments were performed on infections.

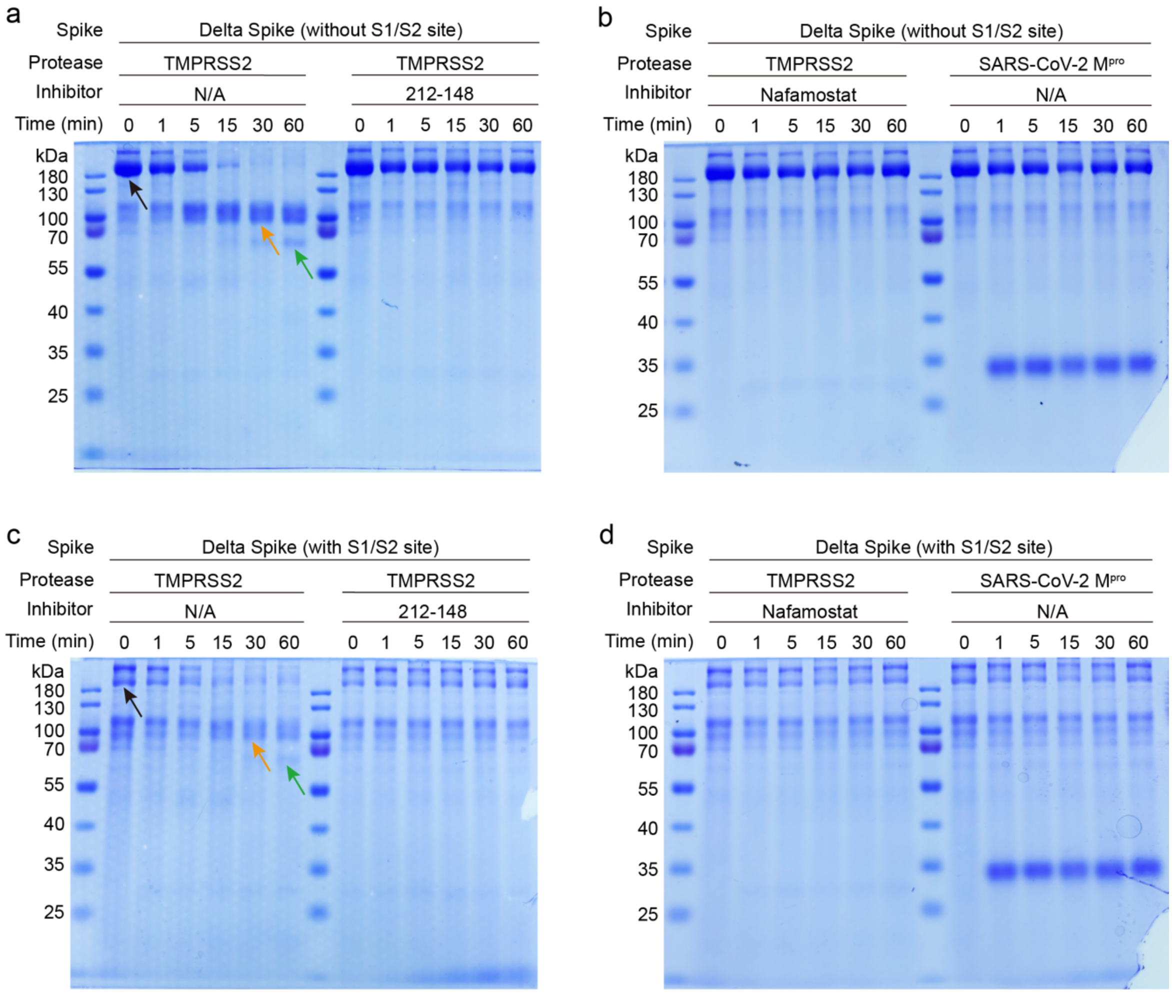

Supplementary Figure 8. Inhibition of TMPRSS2 cleavage of spike protein by 212-148. Time-course cleavage inhibition assay of the mutated spike protein lacking the S1/S2 site (a-b) and original spike protein (c-d) of the SARS-CoV-2 Delta variant. Wherein nafamostat served as a positive control, and SARS-CoV-2 M^{pro} served as a negative control in these assays. Prior to cleavage, TMPRSS2 was preincubated with the 10 times molar 212-148 (15 μM) or nafamostat (15 μM) for 1h. Spike protein is indicated by the black arrows, and cleavage products are indicated by the orange and green arrows.

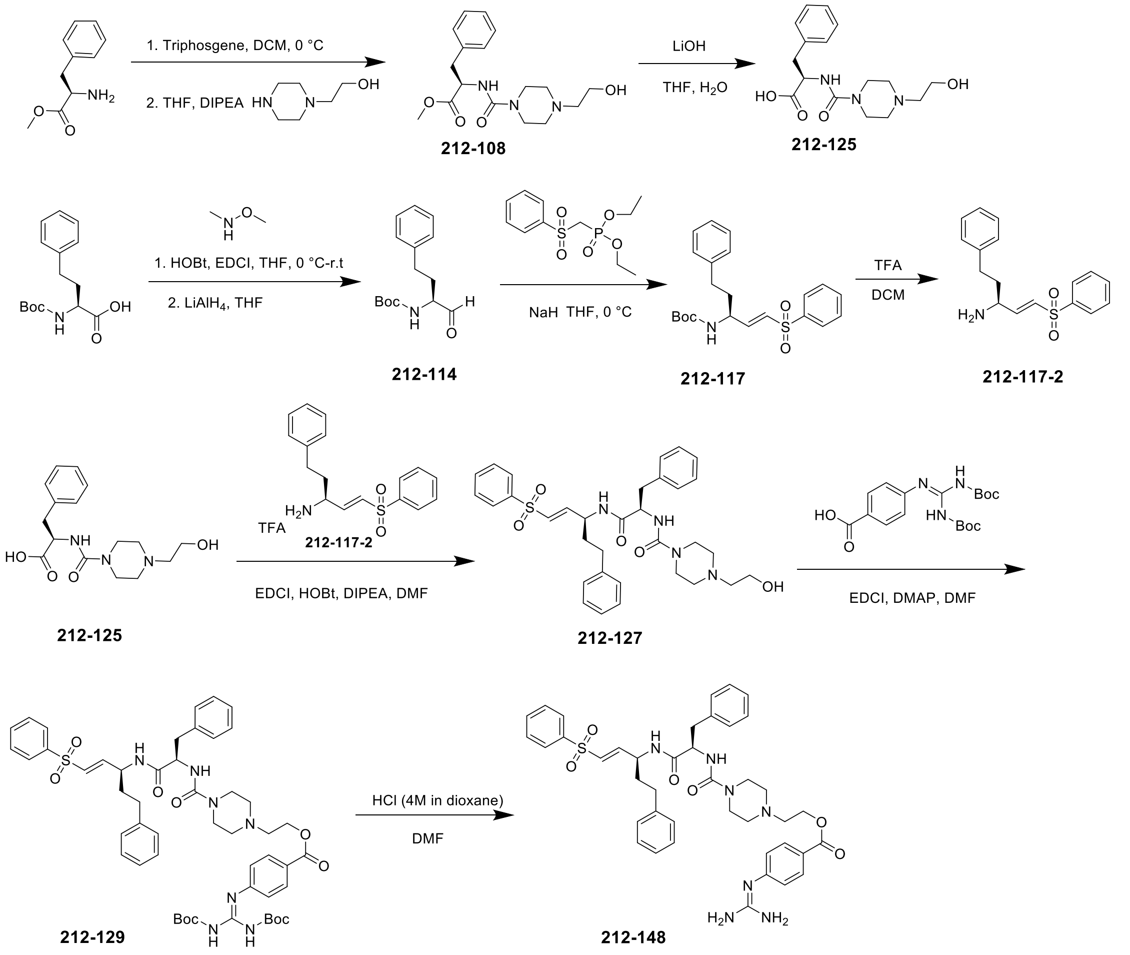

Supplementary Figure 9. The synthesis route of compound 212-148

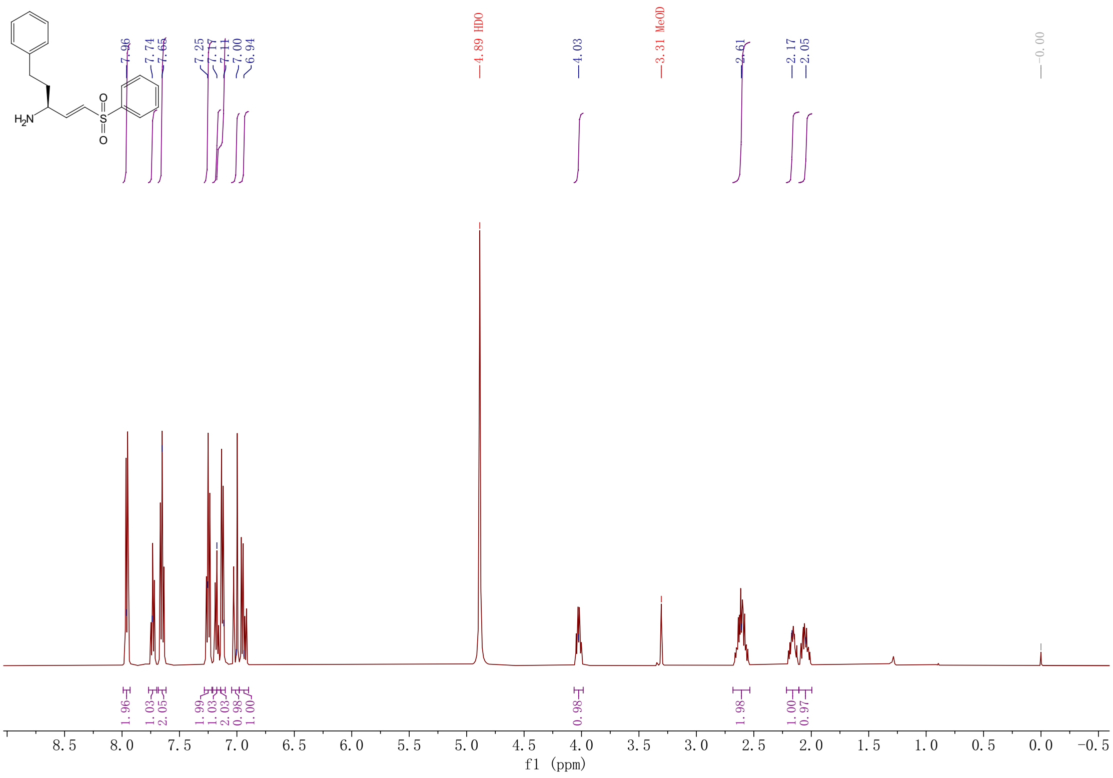

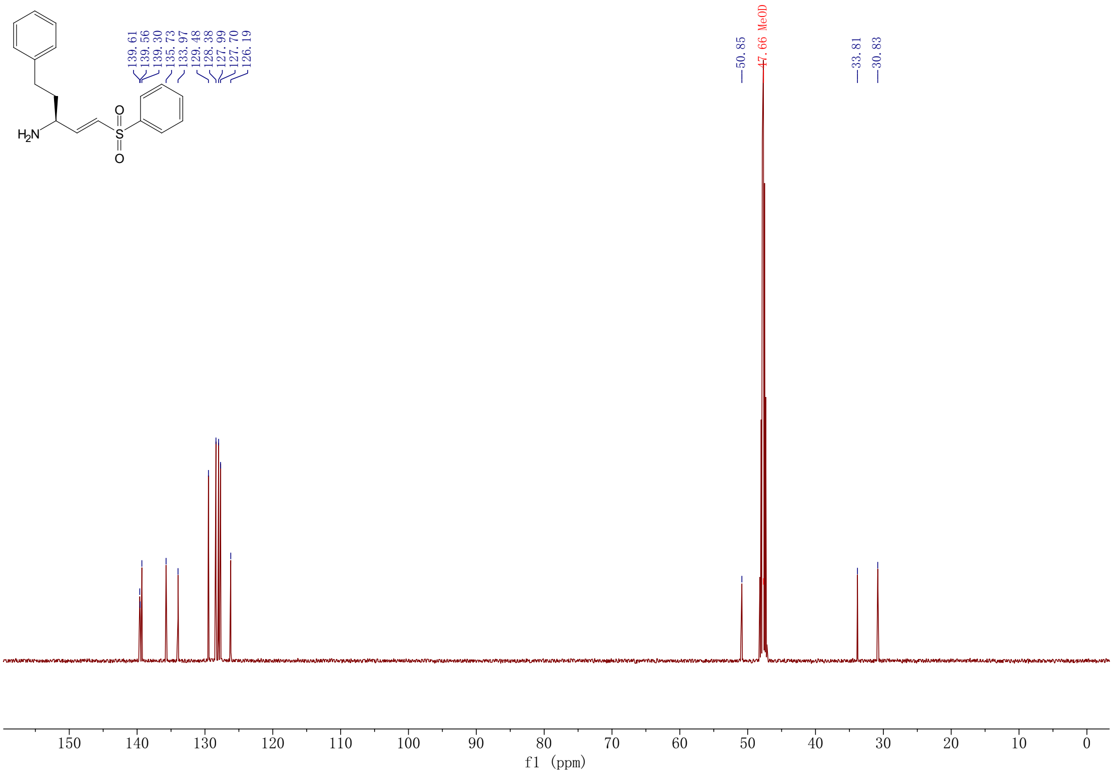

Supplementary Figure 10. NMR spectrum of 212-117-2.

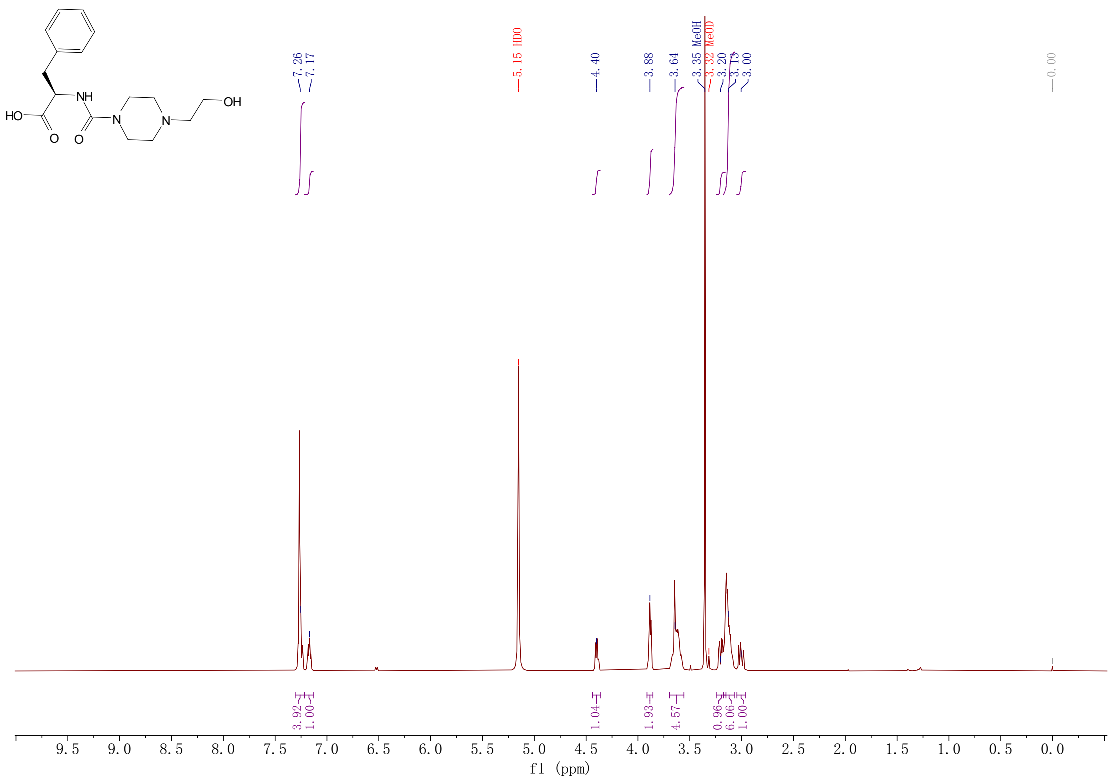

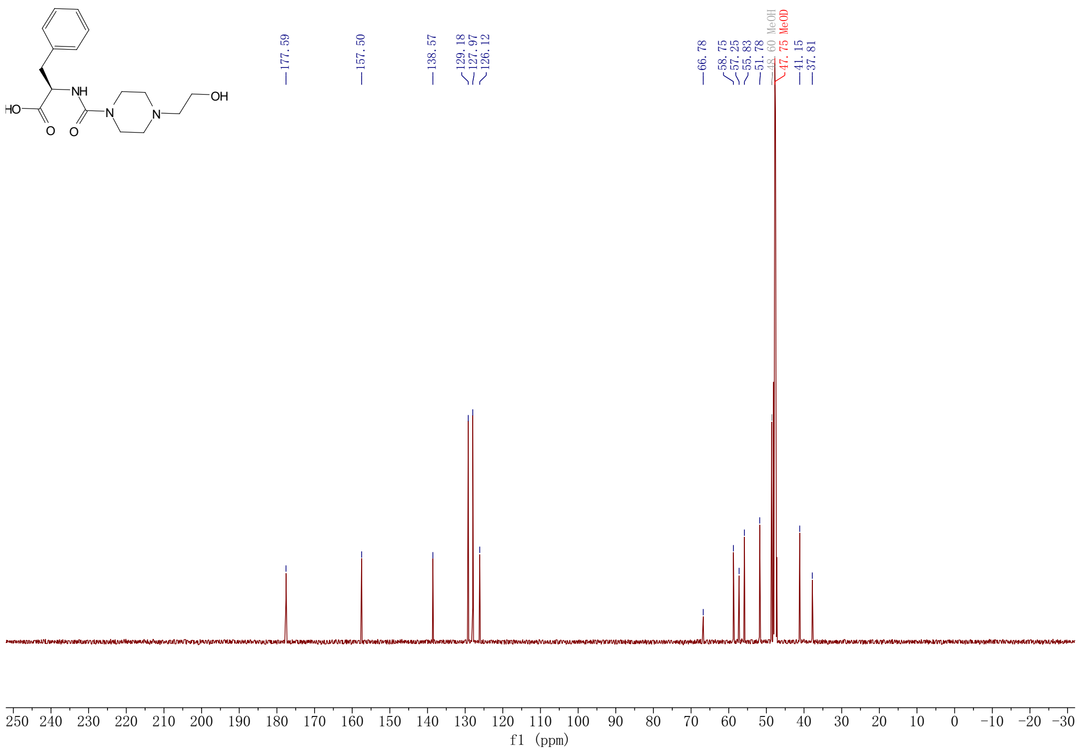

Supplementary Figure 11. NMR spectrum of 212-125.

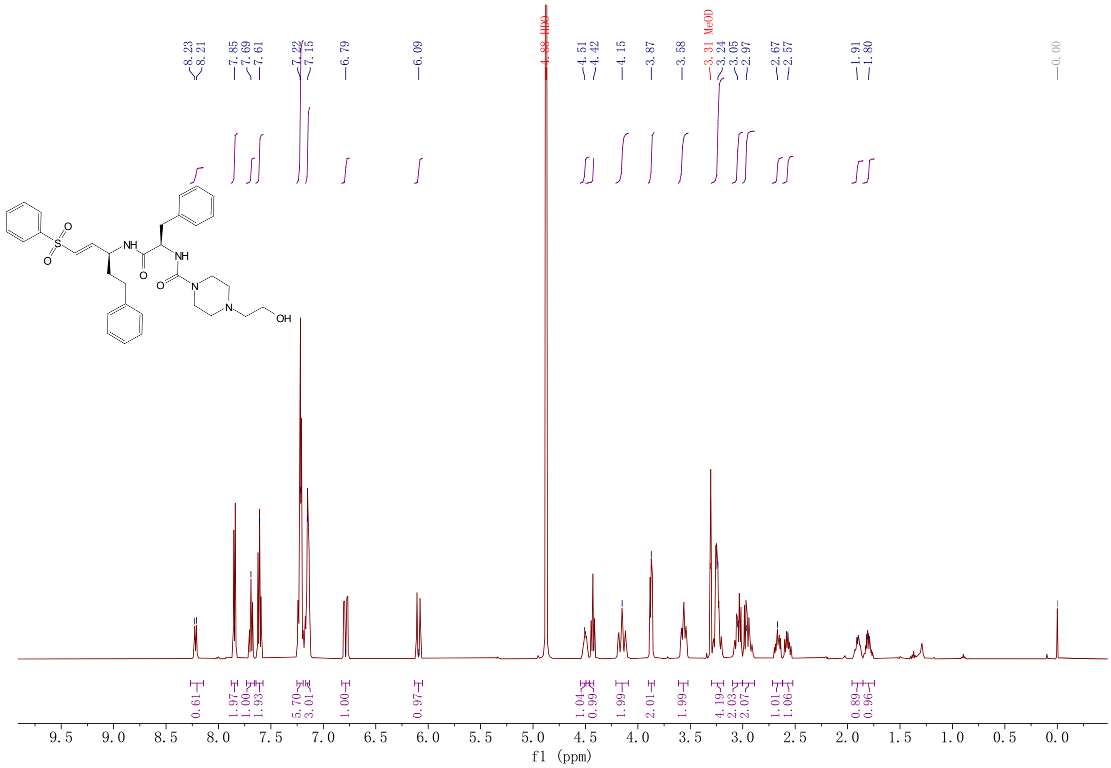

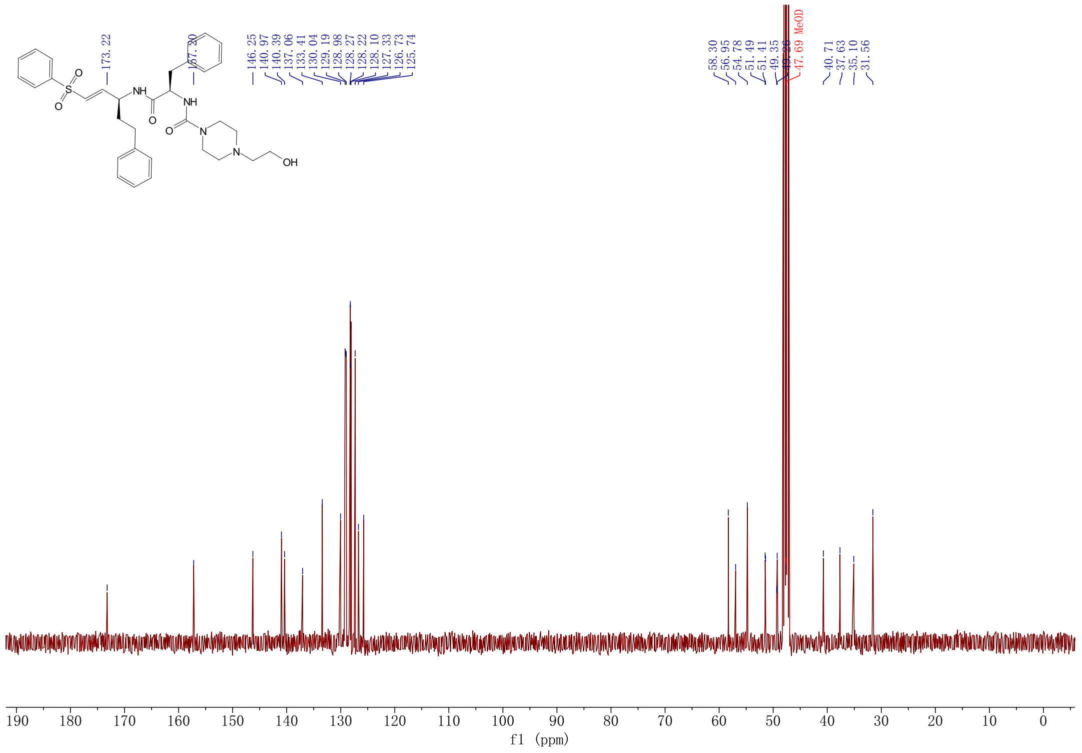

Supplementary Figure 12. NMR spectrum of 212-127.

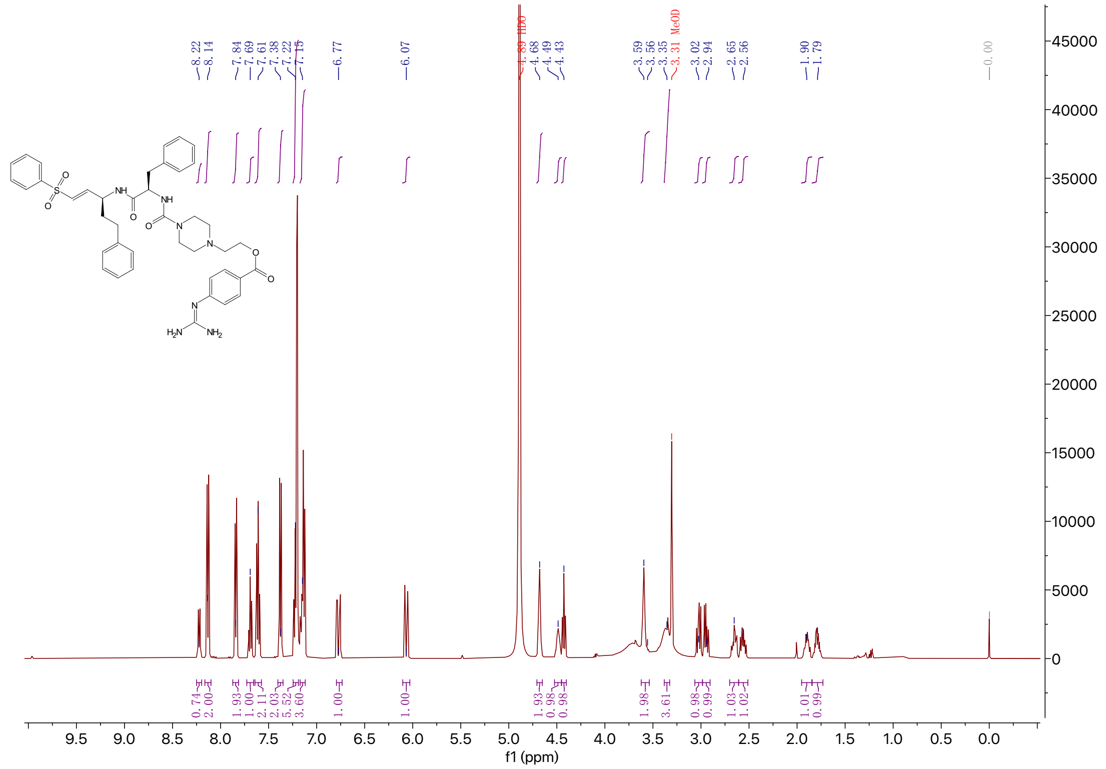

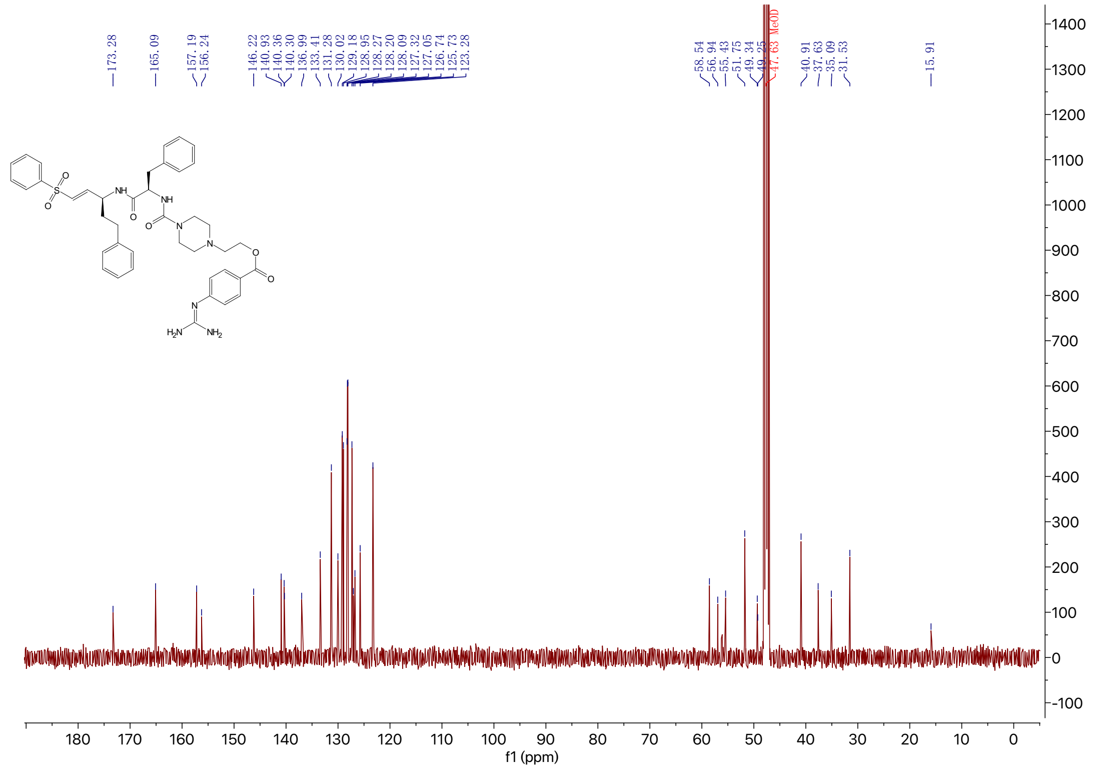

Supplementary Figure 13. NMR spectrum of 212-148.

Supplementary Table 1. X-ray data collection and refinement statistics of crystal structures of CTSB-E64d, CTSB-K777, and CTSB-212-148.

	CTSB-E64d	CTSB-K777	CTSB-212-148
	PDB code: 8HEI	PDB code: 8HE9	PDB code: 8HEN
**Data Collection**			
Space group	$P\,2_1\,2_1\,2_1$	$P\,2_1\,2_1\,2_1$	$P\,2_1\,2_1\,2_1$
Wavelength (Å)	0.9785	0.9537	0.9790
Cell dimensions			
a, b, c (Å)	31.115, 81.509, 94.106	31.432, 70.497, 93.739	31.477, 81.597, 94.687
α, β, γ (°)	90, 90, 90	90, 90, 90	90, 90, 90
Resolution (Å)	30.81-1.55 (1.64-1.55)	39.03-1.55 (1.64-1.55)	37.47-1.95 (2.07-1.95)
No. of unique reflections	34825 (5357)	31146 (4925)	18398 (2854)
Completeness (%)	97.6 (94.6)	99.9 (99.1)	99.5 (98.5)
R_{merge} (%)[a]	14.2 (62.5)	5.7 (87.0)	14.8 (87.1)
Mean $I/\sigma I$	15.92 (4.12)	23.75 (2.65)	12.19 (2.56)
*CC*1/2	99.8 (88.8)	100.0 (95.5)	99.6 (83.9)
Redundancy	10.41 (9.52)	13.26 (13.62)	6.09 (5.21)
Wilson B factors (Å^2)	11.3	21.7	25.5
**Refinement**			
Resolution (Å)	30.81-1.55	39.03-1.55	37.47-1.95
No. of reflections used	34818	31071	18380
R_{work} / R_{free}	15.1/17.2	18.0/20.1	18.5/22.4
No. atoms			
Protein	1954	1941	1947
Ligand/ion	42	57	71
Water	266	130	139
B-factors (Å^2)			
Protein	14.19	34.96	27.97
Ligand/ion	26.55	31.99	36.82
Water	27.48	39.46	33.45
R.m.s. deviations			
Bond lengths (Å)	0.006	0.008	0.008
Bond angles (°)	0.92	1.01	0.97
Ramachandran plot (%)			
Favored (%)	96.84	97.59	96.05
Allowed (%)	3.16	2.41	3.95
Outliers (%)	0	0	0

[a] Values in parentheses are for highest-resolution shell.

[b] $R_{merge} = \sum_h \sum_i | I_{ih} - \langle I_h \rangle | / \sum_h \sum_i \langle Ih \rangle$, where $\langle I_h \rangle$ is the mean intensity of the observations of I_{ih} of reflection h.

[c] $CC_{1/2}$: percentage of correlation between intensities from random half-datasets.

[d] $R\mathrm{work} = \sum_h | F_o - F_c | / \sum_h F_o$, where F_o and F_c are the observed and calculated structure factor amplitudes of reflection h. R_{free} is mathematically equivalent to R_{work}, but was measured over 5% of the data.

Supplementary Table 2. X-ray data collection and refinement statistics of crystal strutures of CTSL-E64d, CTSL-K777, and TMPRSS2-nafamostat.

	CTSL-E64d	CTSL-K777	TMPRSS2-nafamostat
	PDB code: 8HET	PDB code: 8HFV	PDB code: 7XYD
**Data Collection**			
Space group	$P\,2_1\,2_1\,2_1$	$C\ 2$	$P\ 2_1$
Wavelength (Å)	0.9784	0.9785	0.9793
Cell dimensions			
a, b, c (Å)	46.542, 47.705, 100.362	163.150, 38.300, 147.389	49.140, 93.203, 93.130
α, β, γ (°)	90, 90, 90	90, 103.97, 90	90, 100.60, 90
Resolution (Å)	42.22-2.00 (2.12-2.00)	47.68-2.10 (2.22-2.10)	39.80-2.58 (2.74-2.58)
No. of unique reflections	15716 (2483)	52170 (8216)	25667 (4123)
Completeness (%)	100 (99.8)	98.5 (97.4)	98.5 (98.5)
R_{merge} (%)[a]	11.4 (94.4)	18.9 (75.8)	17.1 (84.5)
Mean $I/\sigma I$	17.77 (2.57)	7.47 (2.19)	8.11 (1.88)
*CC*1/2	99.9 (84.5)	99.0 (69.6)	98.9 (64.5)
Redundancy	12.81 (12.53)	5.21 (5.25)	5.29 (5.38)
Wilson B factors (Å^2)	31.29	21.4	39.4
**Refinement**			
Resolution (Å)	42.22-2.00	47.68-2.10	39.80-2.58
No. of reflections used	15707	52146	25657
R_{work} / R_{free}	19.4/21.5	19.7/24.2	21.1/24.8
No. atoms			
Protein	1670	6707	5626
Ligand/ion	24	232	54
Water	170	636	153
B-factors (Å^2)			
Protein	34.00	23.49	45.29
Ligand/ion	32.80	25.06	62.65
Water	38.76	29.28	38.70
R.m.s. deviations			
Bond lengths (Å)	0.006	0.007	0.005
Bond angles (°)	0.90	0.84	0.80
Ramachandran plot (%)			
Favored (%)	97.67	97.68	96.79
Allowed (%)	2.33	2.32	3.21
Outliers (%)	0	0	0

[a] Values in parentheses are for highest-resolution shell.

[b] $R_{merge} = \sum_h \sum_i | I_{ih} - \langle I_h \rangle | / \sum_h \sum_i \langle Ih \rangle$, where $\langle I_h \rangle$ is the mean intensity of the observations of I_{ih} of reflection h.

[c] $CC_{1/2}$: percentage of correlation between intensities from random half-datasets.

[d] $R\text{work} = \sum_h | F_o - F_c | / \sum_h F_o$, where F_o and F_c are the observed and calculated structure factor amplitudes of reflection h. R_{free} is mathematically equivalent to R_{work}, but was measured over 5% of the data.

Supplementary Table 3. X-ray data collection and refinement statistics of crystal structures of TMPRSS2-camostat, TMPRSS2-UK-371804, and TMPRSS2-212-148.

	TMPRSS2-camostat	TMPRSS2-UK-371804	TMPRSS2-212-148
	PDB code: 7Y0E	PDB code: 7Y0F	PDB code: 8HD8
**Data Collection**			
Space group	$P\ 2_1$	$P\ 2_1$	$P\ 2_1$
Wavelength (Å)	0.9792	0.9792	0.9792
Cell dimensions			
a, b, c (Å)	49.157, 93.537, 92.350	48.045, 91.208, 90.696	49.474, 93.835, 94.243
α, β, γ (°)	90, 100.08, 90	90, 100.44, 90	90, 100.13, 90
Resolution (Å)	45.46-2.39 (2.53-2.39)	47.25-2.60 (2.76-2.60)	46.92-2.40 (2.54-2.40)
No. of unique reflections	32630 (5220)	23484 (3715)	33102 (5316)
Completeness (%)	99.5 (99.0)	98.7 (97.5)	99.6 (99.2)
R_{merge} (%)[a]	10.3 (73.5)	16.1 (88.2)	12.0 (70.8)
Mean $I/\sigma I$	10.17 (2.11)	8.48 (1.86)	9.98 (1.71)
*CC*1/2	99.6 (80.6)	98.5 (63.8)	99.5 (61.2)
Redundancy	4.02 (4.12)	3.42 (3.47)	4.41 (3.63)
Wilson B factors (Å^2)	42.0	38.3	38.4
**Refinement**			
Resolution (Å)	45.46-2.39	47.25 -2.60	46.92-2.40
No. of reflections used	32610	23468	33097
R_{work} / R_{free}	19.8/23.7	21.5/23.8	18.3/21.4
No. atoms			
Protein	5582	5552	5684
Ligand/ion	60	80	40
Water	155	92	264
B-factors (Å^2)			
Protein	48.70	43.94	44.82
Ligand/ion	66.63	61.20	58.81
Water	44.86	36.72	40.91
R.m.s. deviations			
Bond lengths (Å)	0.004	0.010	0.005
Bond angles (°)	0.69	1.37	0.77
Ramachandran plot (%)			
Favored (%)	96.96	95.80	96.31
Allowed (%)	3.04	4.20	3.69
Outliers (%)	0	0	0

[a] Values in parentheses are for highest-resolution shell.

[b] $R_{merge} = \sum_h \sum_i | I_{ih} - \langle I_h \rangle | / \sum_h \sum_i \langle Ih \rangle$, where $\langle I_h \rangle$ is the mean intensity of the observations of I_{ih} of reflection h.

[c] $CC_{1/2}$: percentage of correlation between intensities from random half-datasets.

[d] Rwork $= \sum_h | F_o - F_c | / \sum_h F_o$, where F_o and F_c are the observed and calculated structure factor amplitudes of reflection h. R_{free} is mathematically equivalent to R_{work}, but was measured over 5% of the data.
